# Hedgehog inhibitors selectively target cell migration and adhesion of mantle cell lymphoma in bone marrow microenvironment

**DOI:** 10.18632/oncotarget.7320

**Published:** 2016-02-11

**Authors:** Han Zhang, Zheng Chen, Sattva S. Neelapu, Jorge Romaguera, Nami McCarty

**Affiliations:** ^1^ Center for Stem Cell and Regenerative Disease, Brown Foundation Institute of Molecular Medicine for the Prevention of Human Diseases (IMM), The University of Texas-Health Science Center at Houston, Houston, Texas, USA; ^2^ Department of Lymphoma and Myeloma, The University of Texas MD Anderson Cancer Center, Houston, Texas, USA

**Keywords:** mantle cell lymphoma, adhesion, migration, bone marrow microenvironment, hedgehog

## Abstract

The clinical benefits of a Hedgehog (Hh) inhibitor, LDE225 (NPV-LDE-225, Erismodegib), have been unclear in hematological cancers. Here, we report that LDE225 selectively inhibited migration and adhesion of mantle cell lymphoma (MCL) to bone marrows via very late antigen-4 (VLA-4) mediated inactivation of focal adhesion kinase (FAK) signaling. LDE225 treatment not only affected MCL cells, but also modulated stromal cells within the bone marrow microenvironment by decreasing their production of SDF-1, IL-6 and VCAM-1, the ligand for VLA-4. Surprisingly, LDE225 treatment alone did not suppress cell proliferation due to increased CXCR4 expression mediated by reactive oxygen species (ROS). The increased ROS/CXCR4 further stimulated autophagy formation. The combination of LDE225 with the autophagy inhibitors further enhanced MCL cell death. Our data, for the first time, revealed LDE225 selectively targets MCL cells migration and adhesion to bone marrows. The ineffectiveness of LDE225 in MCL is due to autophagy formation, which in turn increases cell viability. Inhibiting autophagy will be an effective adjuvant therapy for LDE225 in MCL, especially for advanced MCL patients with bone marrow involvement.

## INTRODUCTION

Despite several new pharmacological compounds were approved for MCL in the past decade, significant numbers of the patients are still relapsing after treatment. MCL is a highly aggressive subset of B cell Non-Hodgkin's Lymphomas (NHLs) [1]. However since it is relatively minor (~6%) among NHLs and often classified as an orphan disease, the development of compounds that specifically targets MCL has been lacking. Most compounds currently used for MCL treatment were originally developed for leukemia or multiple myeloma.

Hedgehog-mediated signaling has been shown to promote growth and dissemination of several human solid cancers [2–13] as well as tumor growth in hematological malignancies [2–4]. These observations indicate that aberrant activation of the Hh signaling pathway leads to an increase in cell survival and contributes to the maintenance of metastatic behavior. Therefore, targeting the hedgehog pathway by small molecular inhibitors has been attempted in multiple human cancers [5–8]. The most common way to target this pathway is by modulating Smo activities. The most clinically advanced Smo targeting agent is cyclopamine [2, 9–11], and GDC-0449 [7, 9, 12], IPI-926 [13, 14] or GANT61 [9, 15] has been used to block Hh signaling.

LDE225 (NPV-LDE-225, Erismodegib), a new selective and orally bioavailable Smo antagonist, inhibits the Hh signaling pathway via antagonism of the Smo receptor [16–19]. Although LDE225 has been studied in the treatment of many solid tumors and has been shown its effectiveness in inhibiting the Hh pathway [11, 16–19], the effect of LDE225 in hematological malignancies is largely unknown.

We previously discovered PAX5 down-regulated MCL cells are highly drug resistant based on the high throughput screening data of 3864 compounds [20]. Data mining of the compounds identified LDE225 as a potential candidate for inhibiting MCL cell growth and further analyses revealed LDE225 plays a role in migration and adhesion to bone marrow stromal cells (BMSCs). More than 90% of MCL patients have extranodal manifestations and aggressive blastoid types MCL have a high propensity for dissemination and homing to different tissue compartments such as bone marrows [21, 22]. MCL cell trafficking into the microenvironment requires expression and responsiveness of chemokine receptors and adhesion molecules on the lymphoma cells [22]. Although studies have investigated the expression features of chemokine receptors and adhesion molecules in MCL cells [22–26], few agents were found to specifically block cell migration and adhesion of malignant B cells and disrupt the MCL-stroma interaction [27, 28].

We discovered that LDE225 targeted cell migration and adhesion of MCL cells to BMSCs without affecting cell viability. MCL cell infiltration was impaired by LDE225 via very late antigen-4 (VLA-4)-mediated inactivation of focal adhesion kinase (FAK). The inhibition of the Hh signaling pathway by LDE225 also led to decreased stromal cell production of SDF-1 (stromal cell derived factor-1), IL-6 (interleukin-6) and VCAM-1(vascular cell adhesion molecule-1), which further inhibited cell migration via the interaction of SDF-1/CXCR4 and IL-6/IL-6R. Even though cell adhesion and migration were greatly inhibited after LDE225 treatment, MCL cell viability was not affected. This was due to increased CXCR4 levels, which were mediated by reactive oxygen species (ROS). The increased ROS/CXCR4 further enhanced autophagy formation, which in turns increased cell viability.

Taken together, our data provide evidence that LDE225 inhibits cell migration and adhesion through effects on both cancer and stromal cells within the microenvironment. Blocking MCL infiltration to tissues other than lymph nodes may help to prevent MCL progression. We also report, for the first time, that ineffectiveness of LDE225 in MCL is due to autophagy formation, which MCL cells utilized for survival. Autophagy inhibitor 3-methyladenine (3-MA) may be an effective adjuvant therapy for LDE225, especially for those MCL patients who have undergone bone marrow involvement.

## RESULTS

### LDE225 inhibits cell adhesion and migration without suppressing cell viability in MCL cells

We first tested the effects of cyclopamine on cell viability, which demonstrated a low cytotoxicity in MCL cells (Supplementary Figure S1A). This observation was verified using another Hh inhibitor LDE225 (Supplementary Figure S1B). Interestingly, LDE225 dramatically inhibited cell adhesion of MCL cells to BMSCs (Supplementary Figure S1C). Given that advanced MCL cells are dispersed to bone marrows, our results suggest LDE225 could be used to selectively inhibit MCL dispersal. Due to the poor oral bioavailability and acid sensitivity of cyclopamine [29], LDE225, a new hedgehog inhibitor, was selected to further explore the potential biological mechanism of Hh inhibitors in MCL.

**Figure 1:**
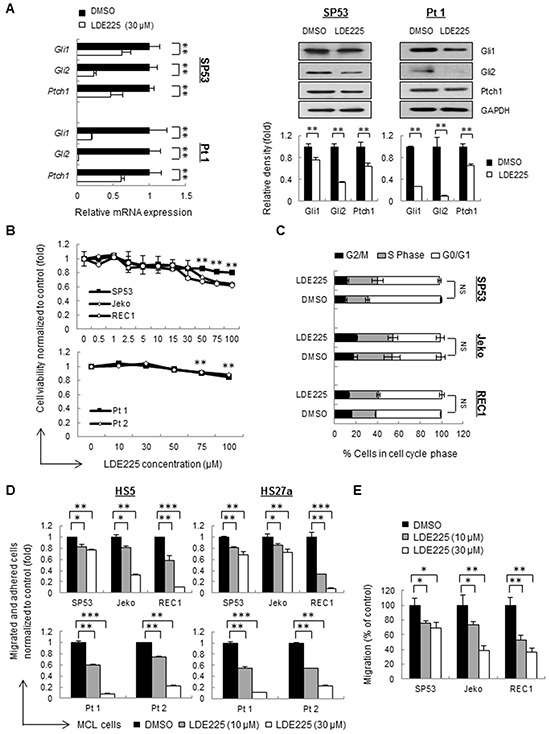
Hedgehog inhibitor LDE225 inhibits cell adhesion and migration without affecting cell viability in MCL cells **A.** The mRNA levels (*Left*) and protein levels (*Right*) of components in the Hh signaling pathway were measured by qRT-PCR and immunoblots. MCL cells (SP53) and primary patient cells were treated with LDE225 (30 μM) or DMSO. Each value in qRT-PCR was normalized to *GAPDH* and represents the mean ± S.D. from three independent experiments. GAPDH was used as a loading control, and the protein levels were quantified using a Gel-Pro Analysis software from three independent immunoblots. **B.** Dose-dependent LDE225-induced cytotoxicity (0-100 μM) at 48 h (primary cells) or 72 h (cell lines) was determined by MTT (3-(4,5-dimethythiazol-2-yl)-2,5-diphenyltetrazolium bromide) assays. Peripheral blood mononuclear cells (PBMC) were isolated from MCL patient aphaeresis blood by standard Ficoll gradient methods and CD19^+^ cells were purified using CD19-MicroBeads (Miltenyi). Data represent the mean ± S.D. from three independent experiments.**p < 0.01 (average viability of LDE225-treated cells vs. average viability of DMSO-treated cells; Student's *t*-test). **C.** Propidium iodide (PI) staining of MCL cells exhibited no obvious changes in the percentages of cells in the S-phase and G0/G1 of the cell cycle after treatment with LDE225 (30 μM). NS, not significant (vs. cells treated with DMSO; Student's *t*-test). **D.** MCL cell adhesion before and after LDE225 treatment (10 μM or 30 μM) was measured using cell lines and patients, which were stained with PKH26 prior to drug treatment. After 72 h of treatment, the cells were seeded onto a pre-established monolayer of HS5 or HS27a BMSCs. PKH26 dye intensity was analyzed and shown as the mean ± S.D. from three independent experiments. **E.** MCL cell migration is significantly reduced after LDE225 treatment. PKH26^+^ MCL cells were treated with LDE225 (10 μM or 30 μM) or DMSO for 72 h and then transferred to the transwell inserts. PKH26 dye intensity of migrated cells in the lower chamber was measured after incubation for 3 hours at 37°C in 5% CO_2_. The percentage of migrated cells relative to the cells treated with DMSO is indicated as the mean ± S.D. *p < 0.05, **p < 0.01, ***p < 1E-05 (vs. cells treated with DMSO; Student's *t*-test).

Firstly, we examined the effects of LDE225 on components of Hh signaling pathways. LDE225 inhibited the expression of the transcription factors Gli1 and Gli2 as well as Ptch1, a downstream target of Gli, suggesting that LDE225 is an effective inhibitor for Hh signaling pathways (Figure 1A, Supplementary Figure S2).

We then analyzed the cell proliferation and viability using different concentrations of LDE225. Proliferation of MCL cell lines as well as cells from MCL patients did not much changed upon treatment, indicating LDE225 didn't affect cell proliferation (Figure 1B). The cell cycle distribution in cell lines also showed no obvious changes in the S-phase and G0/G1 compared to the control cells (Figure 1C).

Interestingly, MCL cells failed to grow in a group after LDE225 treatment even though LDE225 did not affect MCL cell proliferation (Supplementary Figure S3), implying that LDE225 might affect the cell-cell interaction. We selected mid-range concentrations of LDE225 (10 μM or 30 μM) for cell adhesion assays. Pretreatment of LDE225 on MCL cells resulted in a significant decrease in migration and adhesion compared to control cells in a dose-dependent manner (Figure 1D, Supplementary Figure S4). Similarly, cell migration measured using transwell assays also showed that LDE225 dramatically inhibited MCL migration toward the lower chamber (Figure 1E). These findings suggest that LDE225 can effectively block the migration of MCL cells and their adhesion to BMSCs.

### LDE225 inhibits migration and adhesion of MCL cells via VLA-4-mediated inactivation of FAK signaling

**Figure 2:**
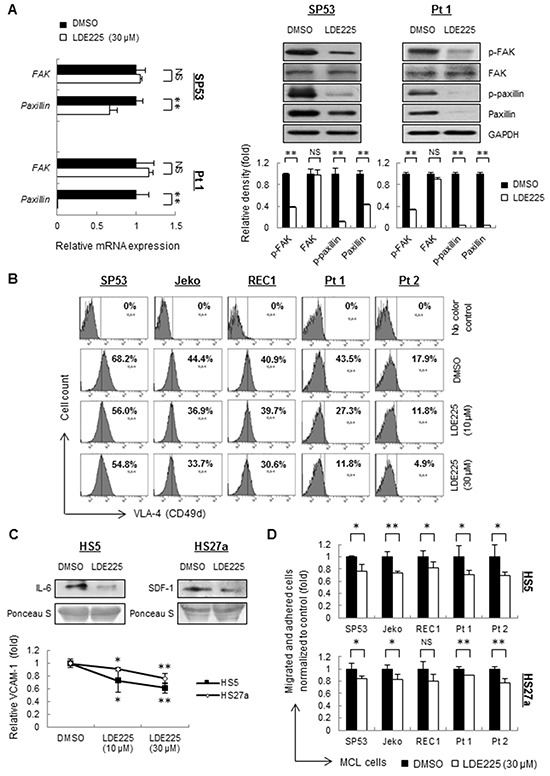
LDE225 inhibits the VLA4-mediated FAK signaling pathway in MCL cells and the production of IL-6, SDF-1 and VCAM-1 in stromal cells **A.** The mRNA levels (*Left*) and protein levels (*Right*) of transducers in the FAK signaling pathway were measured by qRT-PCR and immunoblots. SP53 MCL cells and primary cells were treated with LDE225 (30 μM) or DMSO. Each value in qRT-PCR was normalized to *GAPDH* and represents the mean ± S.D. from three independent experiments. The protein levels were analyzed using of a Gel-Pro Analysis software from three independent immunoblots, and GAPDH was used as a loading control. **B.** Mean fluorescence intensity (MFI) of VLA-4 in MCL cell lines and patient samples were decreased after treatment of LDE225 (10 μM or 30 μM) compared with DMSO in a dose-dependent manner. **C.** Immunoblot analyses of IL-6 (*Top Left*) and SDF-1 (*Top Right*) using conditioned media from HS5 and HS27a stromal cells treated with LDE225 (30 μM). Conditioned media from HS5 and HS27a cells treated with DMSO was used as controls. FACS analysis showed reduced expression of VCAM-1 in HS5 and HS27a cells treated with LDE225. Each value in the diagram was normalized to MFI of VCAM-1 in the cells treated with DMSO (*Bottom*). **D.** MCL cells were stained with PKH26 and were seeded onto the monolayer of HS5 or HS27a BMSCs, which were pre-treated with LDE225 treatment (30 μM) or DMSO for 72 h. PKH26 dye intensity was analyzed and shown as the mean ± S.D. from three independent experiments. NS, not significant,*p < 0.05, **p < 0.01 (vs. cells treated with DMSO; Student's *t*-test).

Lymphocyte migration and adhesion require the cooperation between adhesion molecules and ligands expressed by stromal cells. Studies have shown that FAK signaling pathway is involved in migration and adhesion in human hematological cancers [30, 31], and the integrin expressed on the cell membrane, such as integrin-β4 (ITGB4), VLA-4 or VLA-5, serves as a critical transducer leading to the activation of FAK signaling [15, 32, 33]. To elucidate the molecular mechanisms underlying LDE225-induced cell migration and adhesion in MCL cells, we evaluated the expression levels of FAK and paxillin, a downstream transducer of the FAK signaling pathway. MCL primary cells and cell lines, except for Jeko, demonstrated no change of total FAK in both mRNA and protein levels after LDE225 treatment (Figure 2A, Supplementary Figure S5). However, the phosphorylated FAK, total paxillin and phosphorylated paxillin decreased dramatically in MCL cells treated with LDE225 compared with cells treated with DMSO (Figure 2A, Supplementary Figure S5), suggesting that inhibition of Hh signaling resulted in the disruption of the FAK signaling pathway in MCL cells.

After LDE225 treatment, MCL cells also displayed 20%-73% of reduced VLA-4 expression, which is an upstream signaling of FAK [32], compared to untreated cells (Figure 2B, Supplementary Table S1, Supplementary Figure S6A). These findings demonstrate that the Hh pathway inhibitor LDE225 abrogates the migration and adhesion of MCL cells via VLA-4-mediated inactivation of FAK signaling.

### LDE225 inhibits the stromal cell production of IL-6, SDF-1 and VCAM-1

The decreased migration and adhesion of MCL cells to the BMSCs allowed us to further examine the microenvironment-targeted effects of LDE225. HS5 and HS27a BMSCs, which are reported to produce large amounts of IL-6 and SDF-1, respectively, were used [34]. The levels of IL-6 and SDF-1 produced by BMSCs were largely decreased in both mRNA and protein levels after LDE225 treatment (Supplementary Figure S6B, Figure 2C). Expression of VCAM-1, the ligand for VLA-4, was also decreased (Supplementary Figure S6C, Figure 2C). Pretreatment of LDE225 on HS5 or HS27a cells alone also led to a reduction in migration and adhesion of MCL cells in a co-culture setting (Figure 2D, Supplementary Figure S7) with a low cytotoxicity on cell proliferation (Supplementary Figure S8). These data suggest that Hh inhibition by LDE225 not only affect cell adhesion properties of tumor cells but also can modulate cytokine and chemokine profiles of BMSCs. These collective changes could further affect MCL properties within microenvironment.

### LDE225 inhibits actin polymerization in MCL cells, which reversed by SDF-1 and IL-6

**Figure 3:**
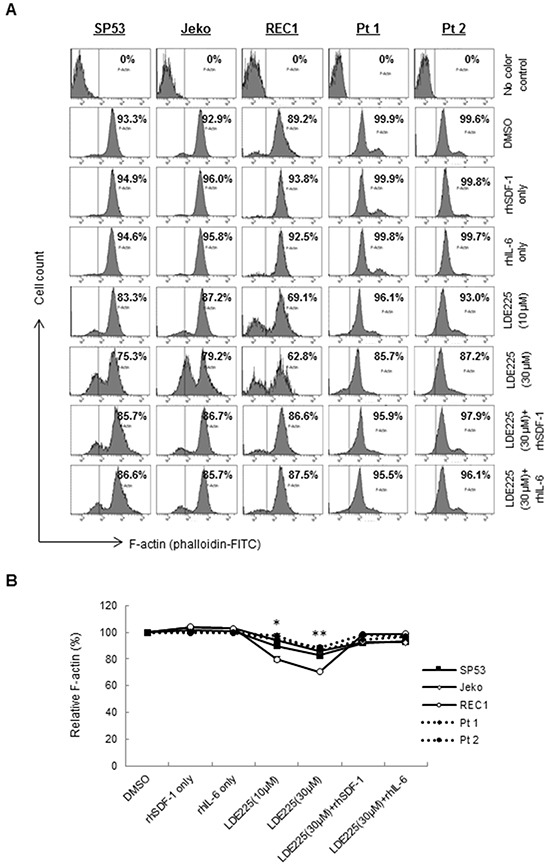
LDE225 inhibits actin polymerization in MCL cells **A.** MCL cells were treated with LDE225 (10 μM or 30 μM) or LDE225 (30 μM) in combination with rhSDF-1 (100 ng/ml) or rhIL-6 (50 ng/ml), and actin polymerization was measured using FITC-phalloidin. Cells treated with rhSDF-1 (100 ng/ml) alone, rhIL-6 (50 ng/ml) alone or DMSO were used as controls. Intracellular F-actin content was determined using FACS analyses. The representative MFI ratio in each sample was indicated. **B.** The relative F-actin content in each treated cell is displayed as the mean ± S.D. from three independent experiments. *p < 0.05, **p < 0.01 (the mean of LDE225-treated cells vs. the mean of DMSO-treated cells from three cell lines and two patient samples; Student's *t*-test).

**Figure 4:**
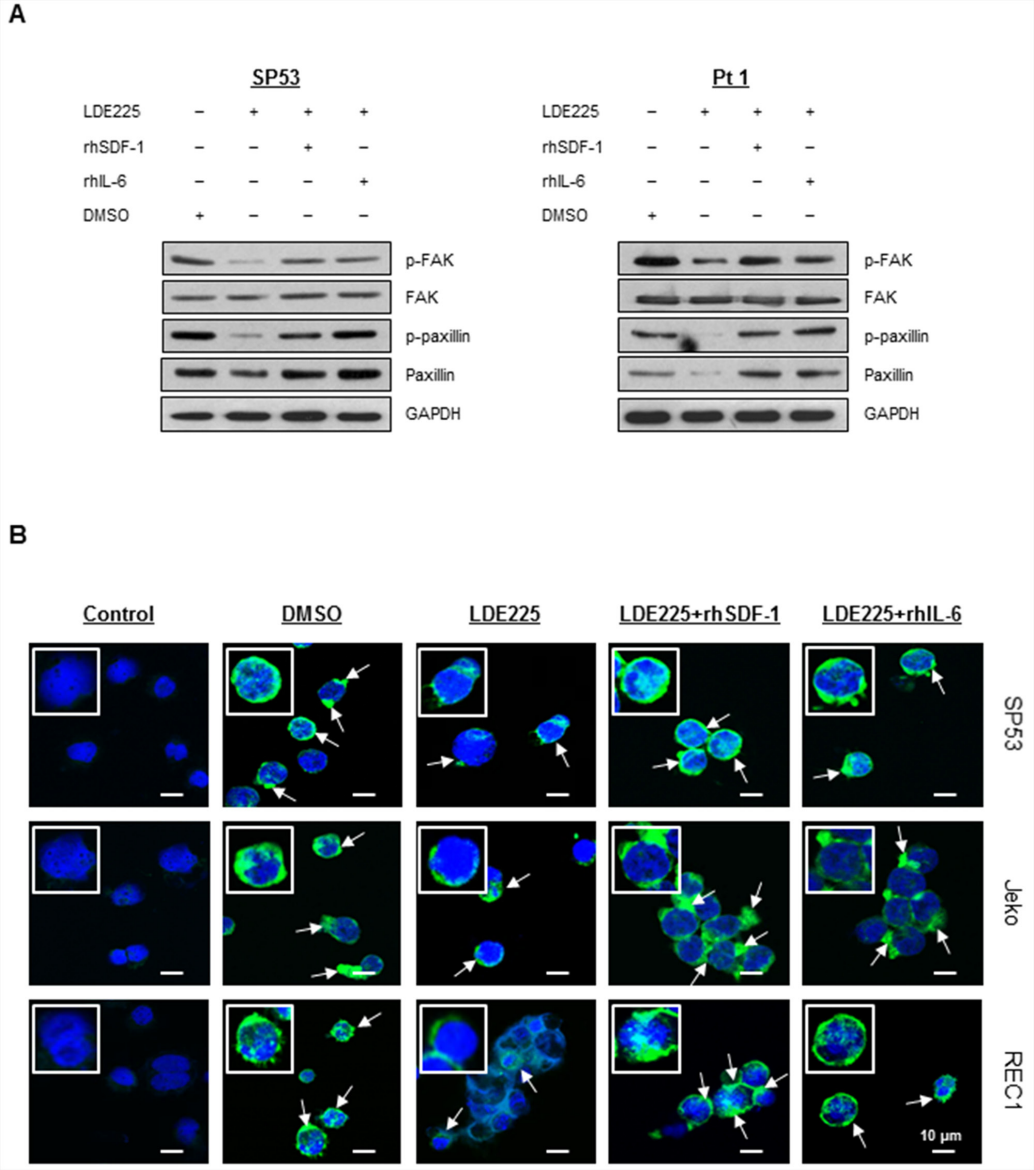
IL-6 and SDF-1 reverse the effect on actin polymerization after LDE225 treatment **A.** Components in the FAK signaling pathway before or after LDE225 treatment (30 μM) with or without SDF-1 (100 ng/ml) and IL-6 (50 ng/ml) were analyzed in the SP53 cells and MCL patient cells using immunoblots. GAPDH was used as a loading control. **B.** Representative confocal microscopic images of MCL cells under different treatments. To visualize F-actin and the nucleus, cells were stained with FITC-phalloidin (green) and draq5 (blue), respectively. F-actin fluorescence (pointed with arrows) in cells treated with LDE225 (30 μM) was reduced compared to DMSO-treated cells. Combination of LDE225 with either SDF-1 (100 ng/ml) or IL-6 (50 ng/ml) reversed F-actin fluorescence to a nearly normal level. Scale bar, 10 μm. Control represents draq-5 stained cells without FITC-phalloidin staining.

Since reorganization of the actin cytoskeleton is an early event in the migration and adhesion of cancer cells [22], we evaluated the ability of LDE225 to induce changes in actin filaments (F-actin). MCL cells were pretreated with LDE225 or DMSO, and then stained with FITC-labeled phalloidin, a toxin that binds specifically to F-actin. Actin polymerization was significantly inhibited by LDE225 in a dose-dependent manner (Figures 3A–3B). We next treated cells with LDE225 combined with recombinant human SDF-1 (rhSDF-1) or IL-6 (rhIL-6). Interestingly, decreased actin polymerization by LDE225 was partially reversed by rhSDF-1 or rhIL-6, suggesting that SDF-1 or IL-6 secreted by BMSCs played an important role in cancer cell migration and adhesion (Figures 3A–3B). This also suggests that LDE225 can be an effective therapy for MCL with extranodal manifestations since it reduces SDF-1 and IL-6 production by stromal cells. To determine the potential signaling pathway of SDF-1 and IL-6 to induce actin polymerization, we evaluated the effects of SDF-1 and IL-6 on the FAK signaling pathway. SDF-1 and IL-6 reversed the LDE225-induced inactivation of FAK signaling, suggesting that SDF-1 and IL-6 modulated cytoskeletal organization via the FAK signaling pathway (Figure 4A).

To further validate the FACS analyses of F-actin, we visualized actin polymerization using fluorescent confocal microscopy. The F-actin staining was shown as an intensity of green fluorescence with FITC-labeled phalloidin (Figure 4B). The F-actin is largely distributed as rings closer to the plasma membrane [35]. LDE225 treatment reduced the thickness and fluorescence intensity of the rings of actin staining compared with cells treated with DMSO. Moreover, LDE225-induced F-actin inhibition was effectively reversed after addition of rhSDF-1 or rhIL-6 (Figure 4B). These findings demonstrate that LDE225 markedly inhibits actin polymerization, leading to the blockade of migration and adhesion; LDE225-induced reduction of chemokine SDF-1 or cytokine IL-6 in BMSCs likely provides supportive blocking of migration within the tissue microenvironment.

### LDE225 upregulates the expression of CXCR4, which is mediated by increased ROS in MCL cells

**Figure 5:**
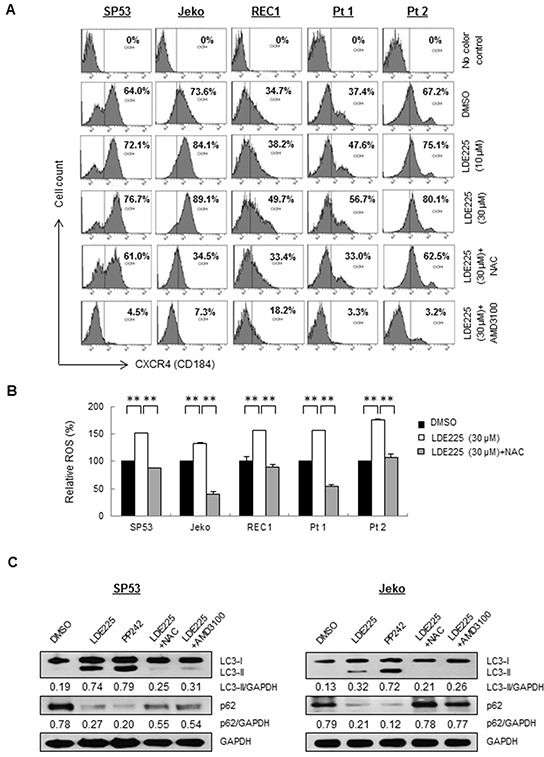
ROS induced CXCR4 stimulates autophagy after LDE225 treatment **A.** MFI of CXCR4 in MCL cell lines and patient cells were increased after treatment of LDE225 (10 μM or 30 μM) compared with DMSO treatment in a dose-dependent manner. Upregulated CXCR4 induced after LDE225 treatment was reduced after ROS inhibitor, NAC (100 μM) or CXCR4 inhibitor, AMD3100 (50 μM) treatment. The MFI values in each sample are as indicated. **B.** ROS production was measured in each cell treated with DMSO or LDE225 (30 μM) or LDE225 (30 μM) combined with NAC using FACS analyses based on DCH-FDA levels. Relative MFI values compared to control are shown. Data represent the mean ± S.D. from three independent experiments. **p < 0.01 (Student's *t*-test). **C.** Inhibition of ROS production with NAC or inhibition of CXCR4 signaling with AMD3100 reduced autophagy induced after LDE225 treatment. SP53 (*Left*) and Jeko (*Right*) cells were treated with LDE225 (30 μM) with 1 h-pretreatment of NAC (100 μM), or combined with AMD3100 (50 μM) for 24 hours. Cells treated with DMSO or autophagy inducer PP242 (1 μM) were used as negative or positive controls. GAPDH was used as a loading control.

Since SDF-1 is an important factor for cancer cell migration to bone marrows, we analyzed the effects of LDE225 on CXCR4, an important chemokine receptor of SDF-1 for MCL homing within the microenvironment [22, 36, 37]. Unexpectedly, after treatment with LDE225 for 72 hours, an increase of *CXCR4* mRNA was observed in MCL cells (Supplementary Figure S6D). CXCR4 protein levels were increased in a dose dependent manner after LDE225 treatment compared to DMSO-treated cells (Figure 5A, Supplementary Table S2).

Since several studies have reported ROS-mediated regulation of CXCR4 in human cancers [37–39], we determined the intracellular level of ROS in MCL cells using FACS. Both MCL cell lines and primary cells showed a statistically significant increase of ROS after LDE225 treatment (Figure 5B). To further explore whether upregulated CXCR4 is mediated by increased ROS effects induced by LDE225, cells were pretreated with ROS antagonist N-acetyl-L-cysteine (NAC) prior to LDE225 treatment. A significant decrease of ROS was observed (Figure 5B). CXCR4 expression on the MCL cell surface was also reduced in NAC-treated cells with approximately 20%-61% reduction compared to the controls (Figure 5A, Supplementary Table S3). Collectively, our data suggest that CXCR4 expression was upregulated in MCL cells mediated by increased ROS induced by LDE225.

### LDE225 induces autophagy for MCL cell survival via increased ROS and upregulated CXCR4

Autophagy, a highly conserved catabolic pathway, plays a pro-survival role in cells under stress such as MCL cells that are resistant to everolimus (RAD001), an mTOR inhibitor [40]. We recently reported that bortezomib treatment induced CXCR4 upregulation and autophagy via ROS in bortezomib-resistant MCL cells [37]. We then treated the cells with LDE225 to test whether autophagy is triggered via a similar pathway. LDE225 treatment led to increased LC3-I to LC3-II conversion, whereas a significant reduction of p62 was observed after LDE225 treatment, indicating increased autophagy formation. Induction of LC3-II was reduced with NAC with increased expression of p62, suggesting NAC treatment decreased autophagy formation (Figure 5C).

**Figure 6:**
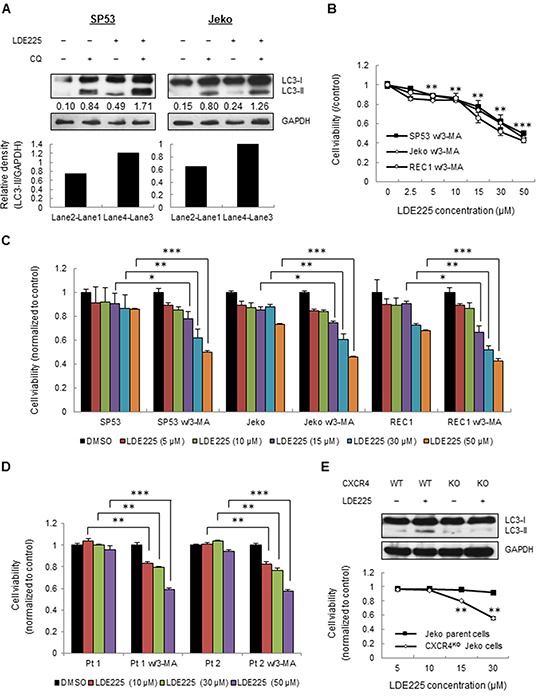
LDE225 increases autophagosome as well as autolysosome formation **A.** SP53 (*Left*) and Jeko (*Right*) cells were treated with LDE225 (30 μM) or DMSO in the presence or absence of lysosomal inhibitor CQ (20 μM) for 24 h. The difference in LC3-II levels in the presence or absence of CQ was larger under LDE225-treated conditions compared with cells under DMSO-treated conditions. Densitometric values of LC3-II were indicated in each lane after normalization to GAPDH using a Gel-Pro Analysis software. Increased values after CQ treatment indicate increased autophagic flux. **B.** Cells were treated with different dosages of LDE225 (0-50 μM) combined with autophagy inhibitor 3-MA (5 mM). The cytotoxicity at 72 h was determined by MTT assays. Data represent the mean ± S.D. from three independent experiments. **p < 0.01, ***p< 1E-05 (average viability of LDE225/3-MA-treated cells vs. average viability of DMSO-treated cells; Student's *t*-test). **C.** Fold changes in cell viability with or without 3-MA treatment are shown after normalization to DMSO-treated cells. Data represent the mean ± S.D. from three independent experiments. *p < 0.05, **p < 0.01, ***p< 1E-05 (vs. cells treated with LDE225 only; Student's *t*-test). **D.** MCL patient cells were treated with LDE225 (0-50 μM) or DMSO in the presence or absence of 3-MA (5 mM). The cytotoxicity at 48 h was determined by MTT assay. Data represent the mean ± S.D. from three independent experiments. **p < 0.01, ***p< 1E-05 (vs. cells treated with LDE225 only; Student's *t*-test). **E.** CXCR4 knockout (CXCR4^KO^) cells were generated using the CRISPR-CAS9 system. LDE225 treatment failed to induce autophagy in CXCR4^KO^ cells (*Top*). Both Jeko parent cells and CXCR4^KO^ Jeko cells were treated under different dosages of LDE225 (5 μM-30 μM). The cytotoxicity at 48 h was determined using MTT assays. Data represent the mean ± S.D. from three independent experiments (*Bottom*). **p < 0.01 (vs. Jeko parent cells; Student's *t*-test).

We next inhibited CXCR4 expression using the CXCR4 antagonist AMD3100 (Figure 5A, Supplementary Table S4). The inhibition of CXCR4 by AMD3100 markedly reduced autophagy formation in MCL cells (Figure 5C), indicating that MCL cells utilize both increased ROS and upregulated CXCR4 signaling to maintain survival via autophagy. To further distinguish whether LC3-II accumulation occurs due to autophagy induction or rather a block in downstream steps, we then performed autophagic flux assays to evaluate autolysosome induction [41]. LC3-II was increased by treatment with the lysosomal inhibitor chloroquine (CQ) under normal conditions (compare lanes 1 and 2); however, the difference in LC3-II levels in the presence and absence of CQ is larger under LDE225 treatment (compare lanes 3 and 4), indicating that autophagic flux was also increased during LDE225 treatment (Figure 6A).

### LDE225 leads to MCL cell death with a combination of autophagy inhibitors

To determine whether LDE225-induced autophagy benefits cell survival in MCL, we treated cells with LDE225 combined with the autophagy inhibitor 3-MA. The combination of LDE225 and 3-MA largely increased cell cytotoxicity compared to LDE225 alone (Figure 6B); LDE225 combined with 3-MA increased cell cytotoxicity more than 40% compared to LDE225 alone (Figure 6C). Similarly, LDE225 increased cell cytotoxicity more than 20%-39% in primary cells when it was combined with 3-MA (Figure 6D).

To further determine the effects of increased CXCR4 signaling on autophagy, we used CXCR4 knockout (CXCR4^KO^) Jeko cells generated by CRISPR-CAS9. The knockout efficiency of CXCR4 was evaluated by FACS analysis (Supplementary Figure S9). Compared to Jeko parent cells, LDE225 treatment failed to induce autophagy formation in CXCR4^KO^ cells (Figure 6E). Notably, CXCR4^KO^ cells were killed by 15 μM and 30 μM LDE225 by more than 15% and 35%, respectively (Figure 6E). Taken together, our data support that LDE225-induced autophagy is an important factor to maintain cell survival via ROS-induced CXCR4 production. Since LDE225 inhibits only cell adhesion and migration, it cannot be used as a sole therapy for MCL. In order to avoid unwanted cancer cell survival resulted from CXCR4 upregulation after LDE225, the drugs that inhibit CXCR4 or autophagy such as AMD3100 or 3-MA can be an effective adjuvant to enhance the cytotoxicity of LDE225.

## DISCUSSION

High grade MCL as well as many metastatic cancers spread to the bone marrows [1, 21, 22, 42, 43]. Even though LDE225 treatment did not affect MCL cell survival, it provides valuable insights on the mechanisms of MCL infiltration to the bone sites. LDE225 was reported to have minimum effects on different hematological malignancies [19, 44], however the mechanisms of those events have not been understood.

Earlier studies have shown that integrin-mediated signaling cascades play important roles in cell adhesion [45–47]. Integrin signaling activates non-receptor tyrosine kinases, including FAK and c-Src, which form a dual kinase complex [45]. FAK kinase activity and signaling are initiated when integrins are clustered in response to chemokines, cytokines or ligand binding [46, 47]. The activated FAK-Src complex further phosphorylates various adaptor proteins, such as paxillin, and promotes cell migration and adhesion by reorganizing F-actin [45]. Several studies have reported that Hh signaling pathway induces cell migration and invasion via FAK signaling in human cancers [15, 48, 49]. In this study, we found that Hh signaling inhibition resulted in a significant decrease of phosphorylation of FAK and paxillin, suggesting that Hh signaling plays a critical role in cell adhesion by inducing FAK phosphorylation. Interestingly, LDE225 inhibited actin polymerization without BMSCs involvement, suggesting that LDE225 can directly modulate actin polymerization or its effects are via a crosstalk between Hh and other signaling pathways.

In addition to its role in cancer cells, we found that blockade of Hh signaling led to decreased production of stromal cell secreted factors (SDF-1, IL-6 and VCAM-1). The migration and adhesion of MCL cells were even blocked after solely treatment of LDE225 on BMSCs. This suggests that Hh inhibition reduces migration and adhesion not only by targeting MCL cells but also by modulating MCL microenvironment. Interestingly, the gene and protein levels of VLA-4 and VCAM-1 were reduced by LDE225, suggesting that the VLA-4/VCAM-1 interaction might be involved in the Hh pathway. Limited studies reported that VLA-4/VCAM-1 is involved in several signaling pathways, such as ERK1/2 or NF-kB [50, 51]; however, no studies have reported the correlation between Hh signaling and VLA-4/VCAM-1 axis. Further studies will be needed to determine whether LDE225 affects the activity of the VLA-4 or VCAM-1 promoter through a direct interaction between Gli transcription factor and VLA-4/VCAM-1 promoters.

To further determine the function of stromal secreted SDF-1 and IL-6 in the migration and adhesion of MCL cells, FAK signaling and actin organization were evaluated in MCL cells. SDF-1 and IL-6 reversed the LDE225-induced inactivation of FAK signaling and actin polymerization to a normal level. These data support that SDF-1 or IL-6 is a potent agent for cell migration and adhesion. Thus, the reduction of SDF-1 or IL-6 induced by LDE225 provides collective effects on blocking of cell migration and adhesion.

Our data also support that unexpected increase of CXCR4 after LDE225 treatment. Interestingly, upregulated CXCR4 was inhibited after blocking ROS, indicating that increased CXCR4 is mediated by ROS. Autophagy is induced under different conditions, including nutrient starvation, intracellular or extracellular oxidative stress, and chemotherapeutic drugs [37, 52–54]. Autophagy formation in LDE225-treated MCL cells was mediated by the upregulation of CXCR4 and increased ROS. Consequently, CXCR4^KO^ Jeko cells were more sensitive to LDE225 compared to parental cells, supporting that both CXCR4 signaling and oxidative stress induced signals are involved in autophagy induction to promote cancer cell survival. Of note, an autophagy inhibitor 3-MA enhanced the cytotoxic effects of LDE225 in MCL cells, suggesting that autophagy confers stress adaptation and promotes viability of MCL under stress such as LDE225 treatment. This finding also suggests why treating LDE225 alone will not be effective despite its effects on cell adhesion and migration. Combined targeting tumor microenvironment, metastasis, and drug resistance may be the only way to eradicate cancer cells [19].

In conclusion, our report is the first to show mechanisms of LDE225 in MCL cells, which inhibits cell migration and adhesion through dual effects on both MCL cells and BMSCs. However, LDE225 treatment resulted in unwanted effects on MCL survival via ROS-induced CXCR4 upregulation and autophagy formation (Supplementary Figure S10). Therefore, it will be important to use LDE225 in combination with other drugs such as autophagy inhibitors, especially for those MCL patients with bone marrow involvement. Understanding of the crosstalk between hedgehog and other signaling pathways in MCL cells will likely provide greater advances in combinatory therapies and improve clinical outcome in MCL patients.

## MATERIALS AND METHODS

### Cell lines

The human mantle cell lymphoma cell lines Jeko and REC1 were obtained from the American Type Culture Collection (Manassas, VA). The human mantle cell lymphoma cell line SP53 was a kind gift from MD Anderson Cancer Center (MDACC). HS5 and HS27a BMSCs were a kind gift from Dr. B. Torok-Storb (Fred Hutchinson Cancer Research Center, Seattle, WA). Cells were maintained at under standard conditions (5% CO_2_, 37°C) and cultured in complete RPMI1640 medium (Cellgro, Manassas, VA) supplemented with 10% fetal bovine serum (FBS), 2 mM L-glutamine, 100≤μg/ml streptomycin and 100 U.I./ml penicillin.

### Human MCL samples

Blood samples from MCL patients were obtained after informed consent, as approved by MDACC as well as by the University of Texas Health Science Center Institutional Review Boards. Mononuclear cells were isolated from all primary patient PBMC using standard Ficoll (Pittsburgh, PA) gradient separation methods.

### Reagents and antibodies

Autophagy inducer PP242 was purchased from Selleck chemicals (Houston, TX). Anti-CXCR4-APC (eBioscience, San Diego, CA, anti-VLA-4-APC (BD Bioscience, San Jose, CA) and anti-VCAM-1-APC (Biolegend, San Diego, CA) were used for FACs analysis. The following antibodies were used for Western blot: anti-Gli1 (Cell Signaling, Danvers, MA), anti-Gli2 (Santa Cruz, Dallas, TX), anti-Ptch1 (Santa Cruz, Dallas, TX), anti-FAK (AbCam, Cambridge, MA), anti-phospho-FAK (Cell Signaling, Danvers, MA), anti-paxillin (BD Biosciences, San Jose, CA), anti-phospho-paxillin (Cell Signaling, Danvers, MA), anti-SDF-1 (Santa Cruz, Dallas, TX), anti-IL-6 (R&D Systems, Minneapolis, MN), anti-LC3 (Novus Biologicals, Littleton, CO), and anti-GAPDH (Cell Signaling, Danvers, MA). Recombinant human SDF-1 (rhSDF-1) was purchased from Pepro Tech (Rocky Hill, NJ), and recombinant human IL-6 (rhIL-6) was purchased from R&D System (Minneapolis, MN). CXCR4 antagonist AMD3100, 2,7-dichlorodihydrofluorescein-diacetate (DCH-FDA, 50MG), ROS inhibitor N-acetyl-L-cysteine (NAC), FITC-labeled phalloidin, l-alpha-lysophosphatidylcholine, and autophagy inhibitor 3-MA were all purchased from Sigma-Aldrich (St Louis, MO).

### Cell adhesion assay

HS5 or HS27a human BMSCs were seeded at a density of 5 × 10^4^ cells per well in a 24-well plate and allowed to form a monolayer overnight. CD19^+^ cells from MCL patients were isolated by magnetic bead-activated cell sorting (MACS) using CD19-MicroBeads following the instructions of the manufacturer (Miltenyi Biotec, Bergisch Gladbach, Germany). MCL cell lines and CD19^+^ primary cells were stained with the membrane dye PKH26 according to the manufacturer's protocol (Sigma, St. Louis, MO). MCL cells were pre-incubated for 72 h with LDE225 (10 μM or 30 μM) or DMSO. Cells were then plated onto the pre-established monolayer of HS5 of HS27a cells (the confluency was approximately 70%) and allowed to adhere for 1 h at 37°C in 5% CO_2_. To investigate the effects of LDE225-treated BMSCs on migration and adhesion of MCL cells, the pre-established monolayer of HS5 or HS27a cells were pre-treated with 30 μM LDE225 or DMSO for 72 h. MCL cell lines and CD19^+^ primary cells stained with PKH26 were then plated onto HS5 or HS27a cells (the confluency was approximately 100%) and allowed to adhere for 1 h at 37°C in 5% CO_2_. After washing with 1 × PBS twice, PKH26 dye intensity was measured using an Infinite®M1000 (TECAN, Morrisville NC) fluorescent plate reader and photographed under microscopy (Olympus IX70, PA, USA). Wells containing just MCL cells with no wash step served as 100% adhesion controls, while wells containing just the monolayer of HS5 or HS27a cells served as 0% adhesion controls.

### Cell viability assay

Cytotoxicity was assessed with Fluorimetric cell viability assay using CellTiter-Blue® (Promega, Madison, WI). Briefly, MCL cells (5 × 10^4^) were incubated in 24-well plates for the indicated times at 37°C with determined doses of drug. After the indicated incubation time, MCL cells were collected and resuspended in RPMI1640; 80 μl of suspended cells were then plated in 96-well plate with 20 μl of CellTiter-Blue® reagents per well, and were further incubated for 2-4 h at 37°C in 5% CO_2_. The fluorescent signal was measured at 560Ex/590Em using a fluorescence plate reader equipped with SoftMax Pro software (Molecular Devices, Sunnyvale, CA), and the level of fluorescent was calculated. Cell viability was assessed based on the value of fluorescent signal of live cells with no drug treatments. Viability of drug treated cells was calculated based on a ratio of the fluorescent signal to that of the non-treated control (DMSO). Blank media only readouts were used as baseline blank controls.

### Cell cycle analysis

Cells (5 × 10^4^) were treated with LDE225 (30 μM) and harvested after cycling for 72 h. Cells were resuspended as a single cell suspension, and were fixed in cold 70% ethanol, added drop wise to the pellet while vortexing. Cells were fixed on ice for 1 h, treated with ribonucleases (RNase A, 100 μg/ml) and stained with 300 μl propidium iodide (PI, 50 μg/ml) for 20 min. When analyzing the cell cycles, cell clumps and duplets were omitted, with only single cells analyzed.

### Cell migration assay

MCL cells (5 × 10^4^) were stained with PKH26, and treated with LDE225 (10 μM or 30 μM) or DMSO for 72 h. Cells were then collected and resuspended in RPMI1640 with no FBS and were added to the Transwell inserts (Falcon, NY, USA) with a pore size of 8 μm. Filters then were transferred to wells containing 10% FBS complete medium. The chambers were incubated for 3 h at 37°C in 5% CO_2_. After this incubation, the PKH26 dye intensity of migrated cells in the lower chamber were measured using an Infinite®M1000 fluorescent plate reader.

### Quantitative real-time PCR (qRT-PCR)

Total RNAs from MCL cell lines and primary MCL cells were extracted using RNAeasy Mini Kit (Qiagen, Valencia, CA). RNA was eluted in DEPC-treated water supplied with kit. Quantification was done by taking absorbance at 260/280 nm ratio using NanoDrop (Eppendorf BioPhotometer plus, Hamburg, Germany). Equal amounts of RNA were used for cDNA synthesis using SuperScript II Reverse Transcriptase (Invitrogen, Carlsbad, CA). cDNA was diluted 1:5 in DEPC-treated water prior to PCR amplification. The involved genes and primers are shown in Supplementary Table S5. qRT-PCR was performed using SYBR Green/ROX MasterMix Plus (Qiagen, Valencia, CA) on an ABI-7000 (Applied Biosystems, Foster City, CA). The reaction was performed with a starting temperature of 95°C for 10 min, followed 40 cycles of 15 s at 95°C, 30 s at 60°C, and 30 s at 72°C. *GAPDH* gene was used as an internal control. The relative expression level of each gene was normalized to the *GAPDH* by the method of 2^−ΔΔCt^.

### Immunoblotting and semi-quantitative analysis

To examine Hh pathway, MCL cell lines or CD19^+^ primary cells were pretreated with LDE225 (30 μM) or DMSO for 72 h or 48 h, and then lysed. To examine FAK signaling pathway, MCL cells were pretreated with LDE225 (30 μM) or DMSO for 72 h with or without adding rhSDF-1/IL-6 for the last 5 min, and were then transferred onto the pre-established monolayer of HS27a cells for 20 min at 37°C in 5% CO_2_ [47]. After this incubation, the supernatant cells were collected. Cells adhered to stromal cells were gently washed and collected without disturbing BMSCs. For autophagy assays, MCL cells were treated with LDE225 (30 μM) or DMSO for 24 h with or without 1 h-pretreatment of NAC (100 μM), co-treatment with AMD3100 (50 μM) or lysosomal inhibitor CQ (20 μM). Total harvested cells were lysed to perform immunoblotting assay, as previously described [38]. Immunoblotting was subjected to semi-quantitative analysis using Gel-Pro Analyzer 4.0 software. The relative expression levels of proteins were normalized to the integrated optical density (IOD) of proteins compared with GAPDH (loading control).

### Flow cytometry analysis

Cells were collected and stained with specific saturating antibody concentration for 20 min on ice in PBS with 2% FBS and 1% streptomycin/penicillin (FACS buffer), and then wash two times. Cells were resuspended in FACS buffer with DAPI. Live cells were gated based on DAPI expression. MCL primary cells were gated based on their CD19 expression. FACS was performed on a BD FACS Aria II system. Data were analyzed using the provided FACS Diva software.

### Actin polymerization

MCL cells (10^6^ cells) were collected after pretreatment of LDE225 (10 μM or 30 μM) or DMSO for 72 h with or without adding rhSDF-1 or rhIL-6 (5 min treatment prior to harvest). Cells were washed twice with FACS buffer. Resuspended cells in 400 μl FACS buffer, and added 100 μl of staining solution containing 0.4 μM FITC-labeled phalloidin, 0.5 mg/ml l-alpha-lysophosphatidylcholine, and 3.7% formaldehyde in water for 20 min at 37°C. The fixed cells were analyzed by flow cytometry, and results were plotted relative to the mean fluorescence intensity (MFI) of the cells with DMSO treatment.

### Assessment of reactive oxygen species (ROS)

LDE225-induced intracellular generation of ROS in MCL cells was assessed by employing a specific cell permeable fluorescent probe, DCH-FDA. Briefly, MCL cells (2 × 10^5^ cells) were incubated in complete medium with DMSO or LDE225 (30 μM) for 72 h, followed by labeling of cells with DCH-FDA (10 μM) in FACS buffer for 20 min at 37°C in the dark. The samples were measured at Ex/Em, 480/520 nm and then analyzed by flow cytometry. Results were plotted relative to the MFI of the cells with DMSO treatment.

### Confocal microscopy and image analysis

MCL cells were collected after pretreatment of LDE225 (30 μM) or DMSO for 72 h with or without adding rhSDF-1 or rhIL-6 (5 min treatment prior to harvest). Cells were washed with ice-cold PBS, and stained with FITC-labeled phalloidin as described above. An aliquot (100 μl, 5 × 10^3^ cells) of cell suspension was layered onto glass slides at 800 rpm for 15 min (Cytospin 4, Thermo Scientific). Draq5™ (Abcam, Cambridge, MA) was used to counter stain the nucleus. Each slide was examined for the presence of F-actin and Draq5 (nucleus) at 488 nm and 633 nm excitations and the data were compared pixel by pixel. Image acquisition of each slide was done at the same parameters of confocal microscopy using sequential mode for F-actin (green) and Draq5 (blue).

### Statistical analysis

Data reported are expressed as experimental mean ± standard deviations. Statistical significance of differences between control and experimental groups was evaluated by the Student t-test, where p < 0.05 was considered statistically significant. All experiments and assays were repeated at least three times and performed in duplicate or triplicate.

## SUPPLEMENTARY FIGURES AND TABLES


