# Supplementary material for: Hedgehog inhibitors selectively target cell migration and adhesion of mantle cell lymphoma in bone marrow microenvironment
Source: Oncotarget. 2016 Feb 11;7(12):14350–65. doi: 10.18632/oncotarget.7320 (PMC4924720; doi:10.18632/oncotarget.7320)
Supplement: Supplementary file 1 [file oncotarget-07-14350-s001.pdf]

SUPPLEMENTARY FIGURES AND TABLES

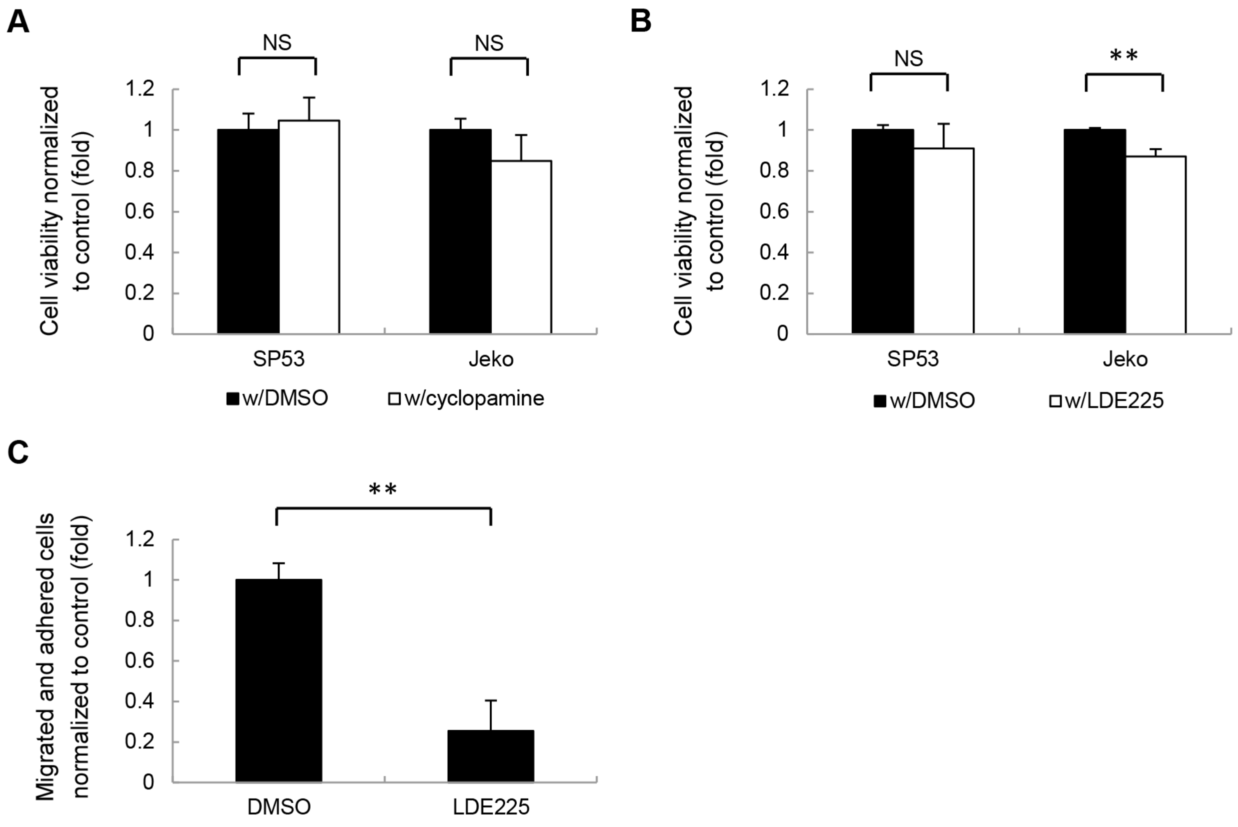

Supplementary Figure S1: Hedgehog inhibitors cyclopamine **A.** and LDE225 **B.** induced cytotoxicity (10 µM) at 72 h in SP53 and Jeko cells was determined by MTT assays. Data represent the mean ± S.D. from three independent experiments. **C.** Cell adhesion screenings after LDE225 treatment (30 µM) was measured in Jeko cells, which were stained with PKH26 prior to drug treatment. After 72 h-treatment, the cells were seeded onto a pre-established monolayer of HS5 bone marrow stromal cells. PKH26 dye intensity was analyzed and shown as the mean ± S.D. from three independent experiments. NS, not significant, **$p < 0.01$ (vs. cells treated with DMSO; Student's *t*-test).

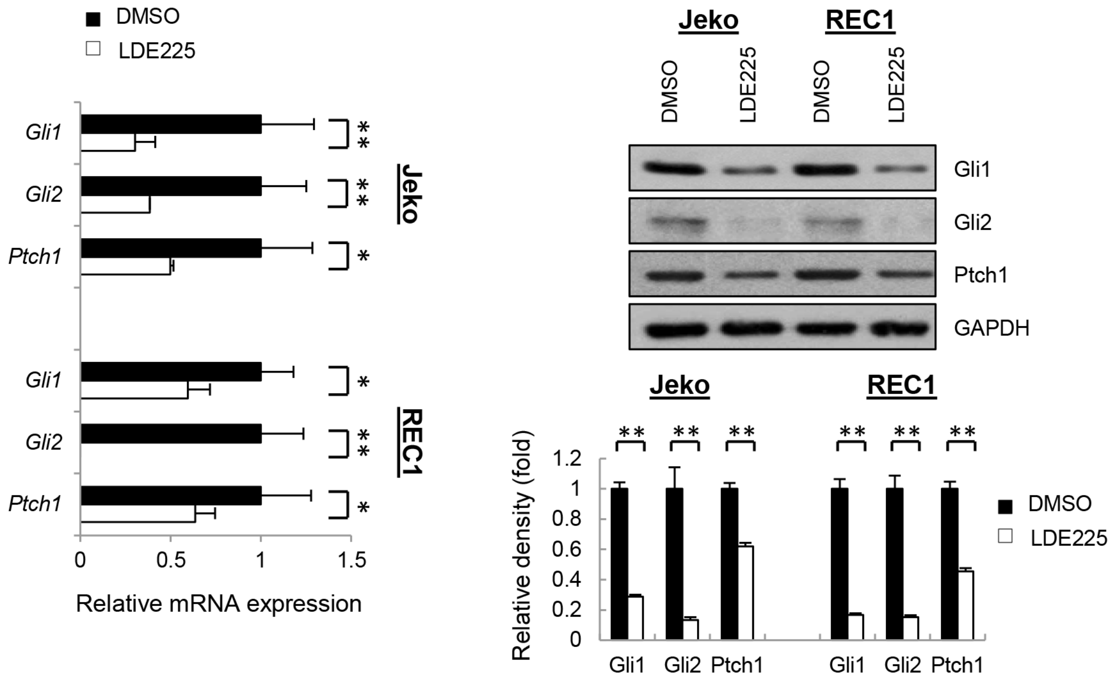

Supplementary Figure S2: The mRNA and protein levels of components in the Hh signaling pathway were measured by RT-PCR and Western blot in Jeko and REC1 MCL cell lines after LDE225 treatment (30 μM) or DMSO as a control. Each value in qRT-PCR was normalized to *GAPDH* and represents the mean ± S.D. from three independent experiments. The protein levels were semi-quantified by analysis of the Western blot with Gel-Pro Analysis software from three independent immunoblots, and GAPDH was used as a loading control. *p < 0.05, **p < 0.01 (vs. cells treated with DMSO; Student's *t*-test).

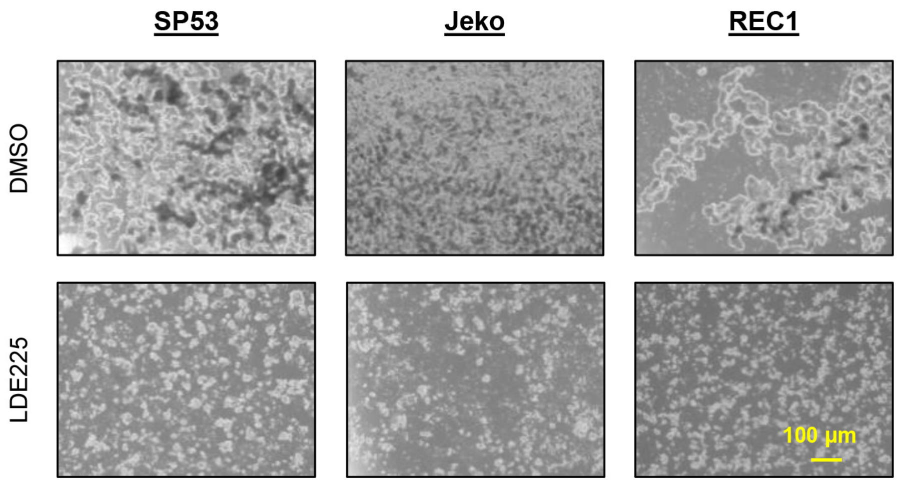

Supplementary Figure S3: MCL cells from SP53, Jeko and REC1 were treated with LDE225 (30 μM) or DMSO. After treatment, LDE225 treated cells were resistant to group with each other compared with cells treated with DMSO. The plates were photographed under microscopy. Scale bar, 100 μm.

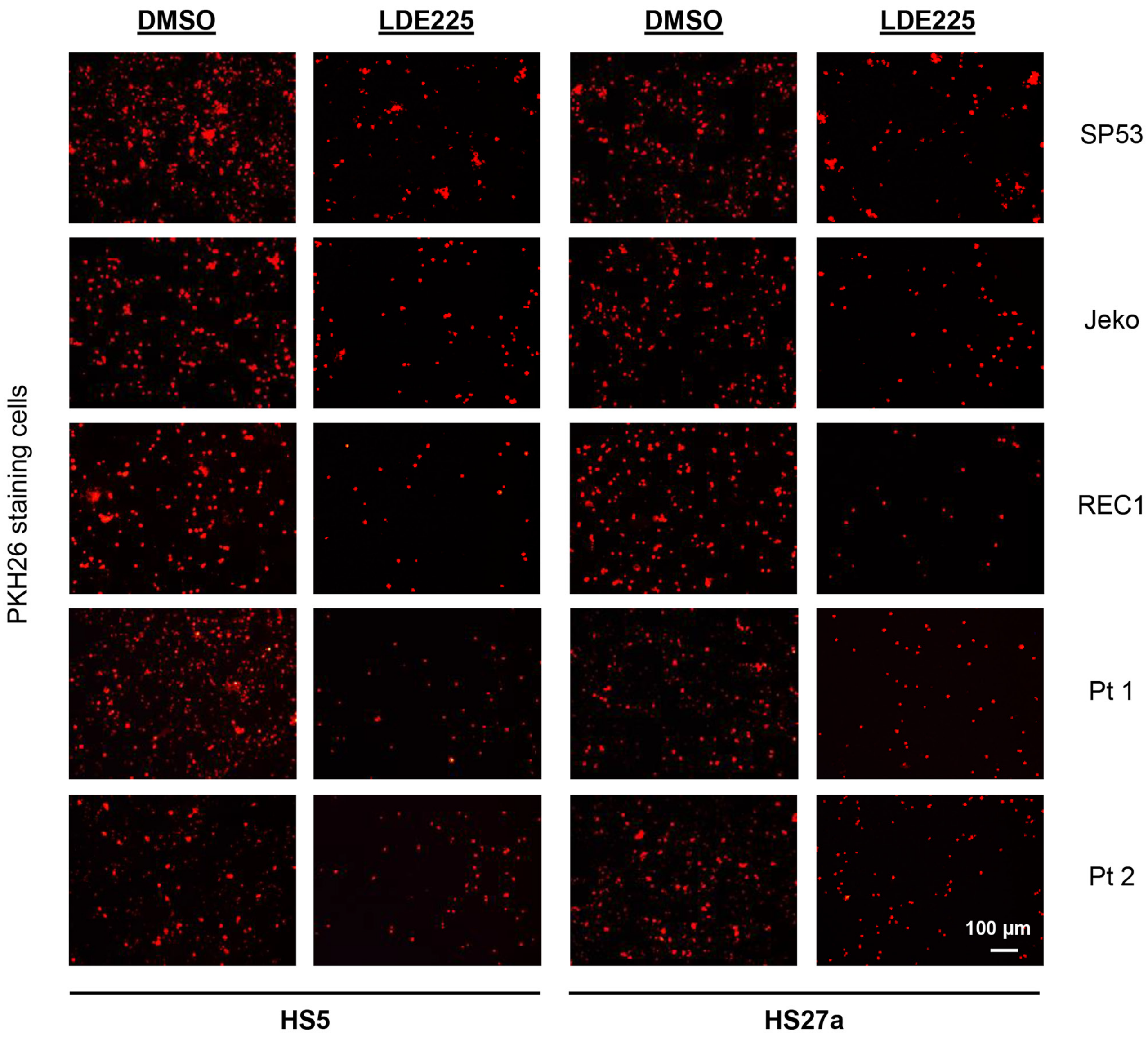

Supplementary Figure S4: Representative microscopic images of adhered MCL cells in a co-culture setting. MCL cells from three cell lines and two patients were stained with PKH26 and subsequently treated with LDE225 (30 µM) or DMSO. After 72 h of treatment, cells were seeded onto a pre-established monolayer of HS5 or HS27a bone marrow stromal cells. The plates were photographed under microscopy. Scale bar, 100 µm.

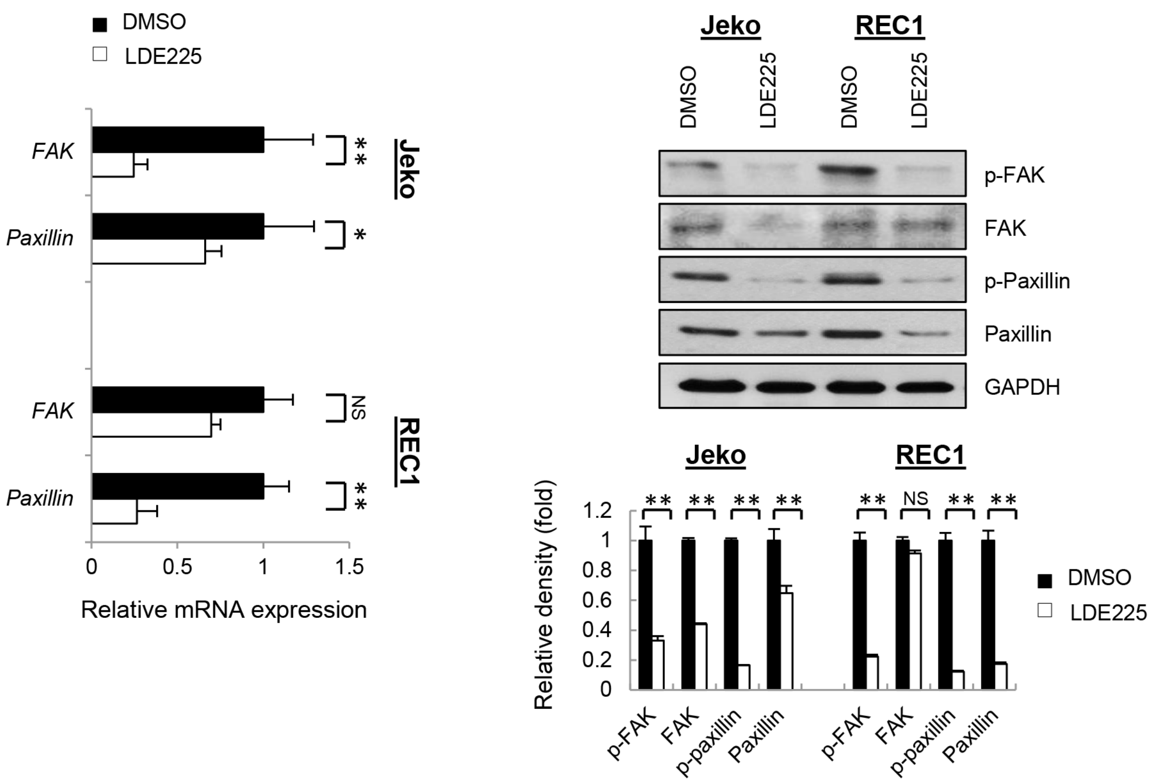

Supplementary Figure S5: The mRNA and protein levels of transducers in the FAK signaling pathway were measured by RT-PCR and Western blot in Jeko and REC1 MCL cell lines after LDE225 treatment (30 μM) or DMSO as a control. Each value in qRT-PCR was normalized to *GAPDH* and represents the mean ± S.D. from three independent experiments. The protein levels were semi-quantified by analysis of the Western blot with Gel-Pro Analysis software from three independent immunoblots, and GAPDH was used as a loading control NS, not significant, $*p < 0.05$, $**p < 0.01$ (vs. cells treated with DMSO; Student's *t*-test).

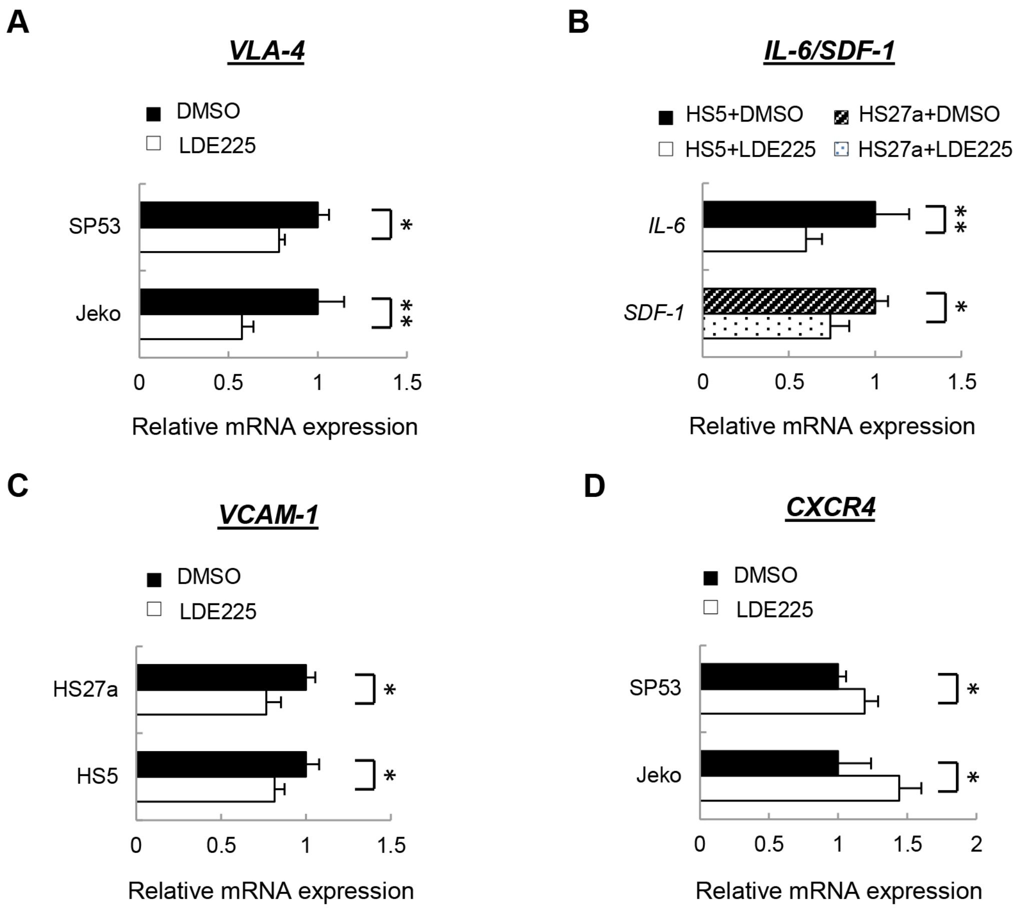

Supplementary Figure S6: A. *VLA-4* mRNA levels were measured by qRT-PCR in SP53 and Jeko MCL cells treated with LDE225 (30 µM) or DMSO. **B.** *IL-6* mRNA levels in HS5 stromal cells and *SDF-1* mRNA levels in HS27a stromal cells were detected by qRT-PCR with LDE225 treatment (30 µM) or DMSO. **C.** *VCAM-1* mRNA levels in both HS5 and HS27a stromal cells were measured by qRT-PCR with LDE225 treatment (30 µM) or DMSO. **D.** *CXCR4* mRNA levels were measured by qRT-PCR in SP53 and Jeko MCL cells treated with LDE225 (30 µM) or DMSO. Each value in qRT-PCR was normalized to *GAPDH* and represents the mean ± S.D. from three independent experiments. *p < 0.05, **p < 0.01 (vs. cells treated with DMSO; Student's *t*-test).

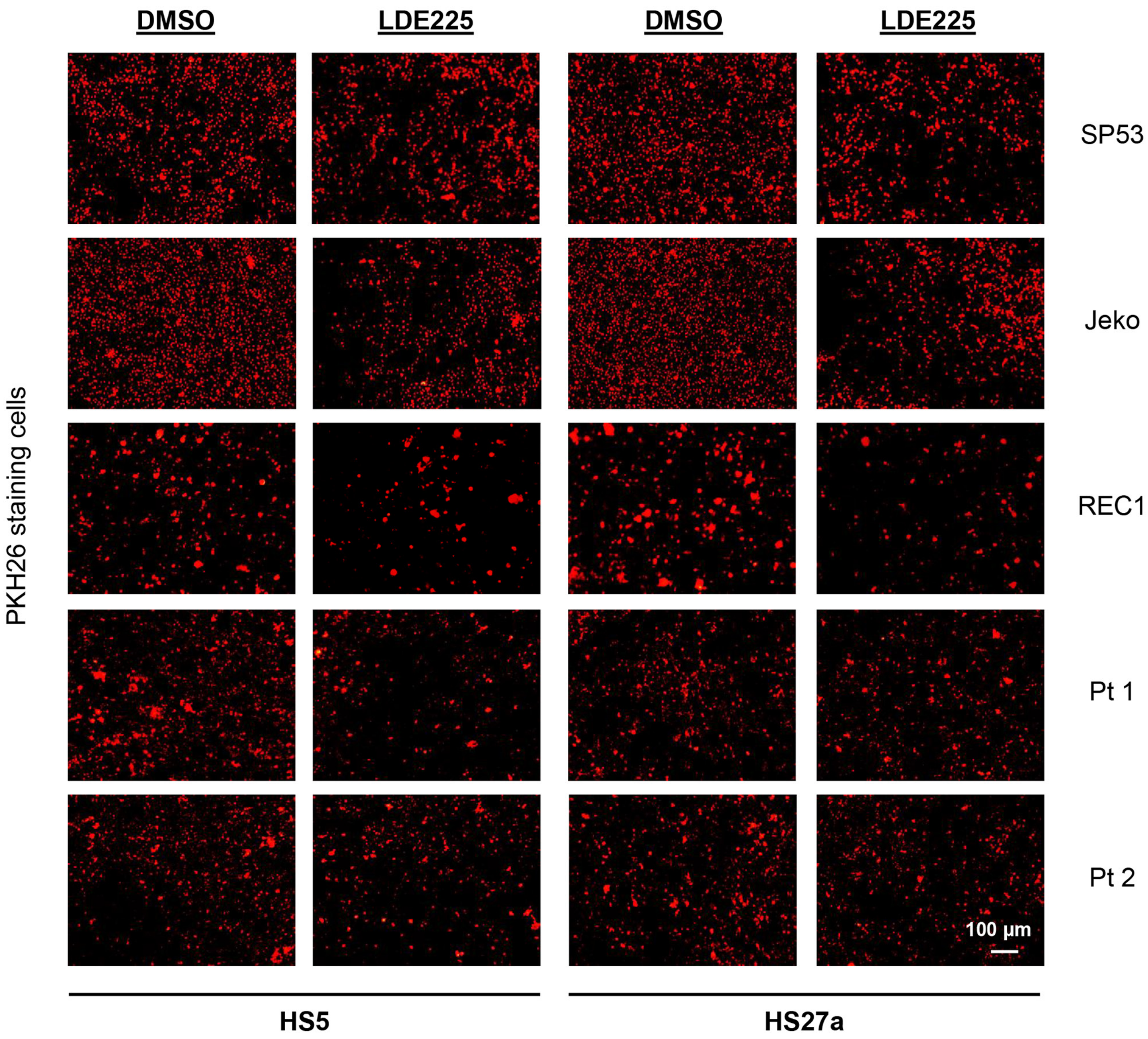

Supplementary Figure S7: Representative microscopic images of adhered MCL cells in a co-culture setting. The monolayer of HS5 or HS27a cells was treated with LDE225 (30 µM) or DMSO for 72 h. MCL cells from three cell lines and two patients were stained with PKH26 and were subsequently seeded onto pre-treated HS5 or HS27a cells. The plates were photographed under microscopy. Scale bar, 100 µm.

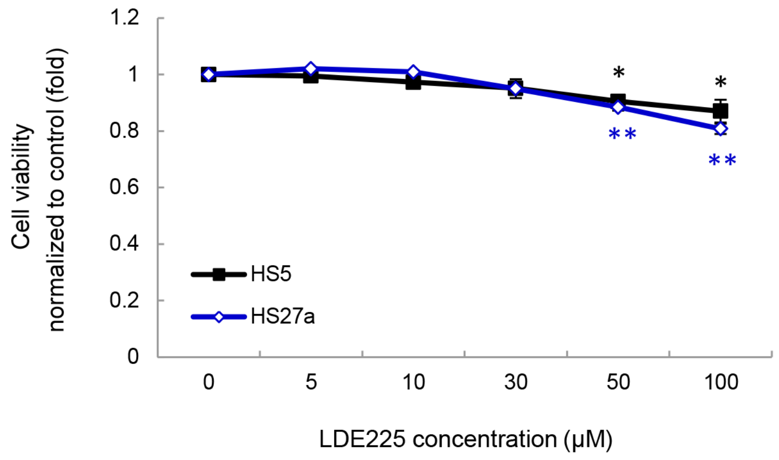

Supplementary Figure S8: Dose-dependent LDE225-induced cytotoxicity (0-100 µM) in HS5 (black) or HS27a (blue) cells at 72 h was determined by MTT assays. Data represent the mean ± S.D. from three independent experiments. $^*p < 0.05$, $^{**}p < 0.01$ (black: HS5 cells with LDE225 vs. cells with DMSO; blue: HS27a cells with LDE225 vs. cells with DMSO; Student's *t*-test).

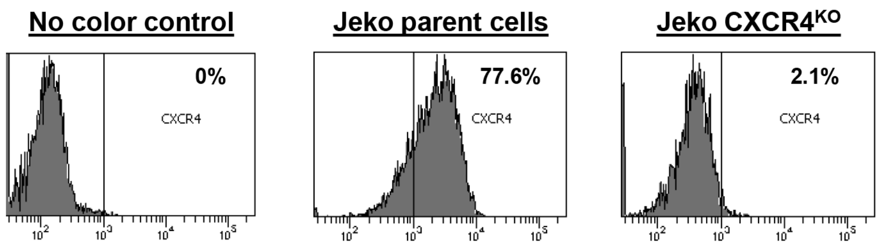

Supplementary Figure S9: Mean fluorescence intensities (MFI) of CXCR4 in Jeko parent cells and CXCR4^{KO}cells were detected. The MFI values in each sample are as indicated.

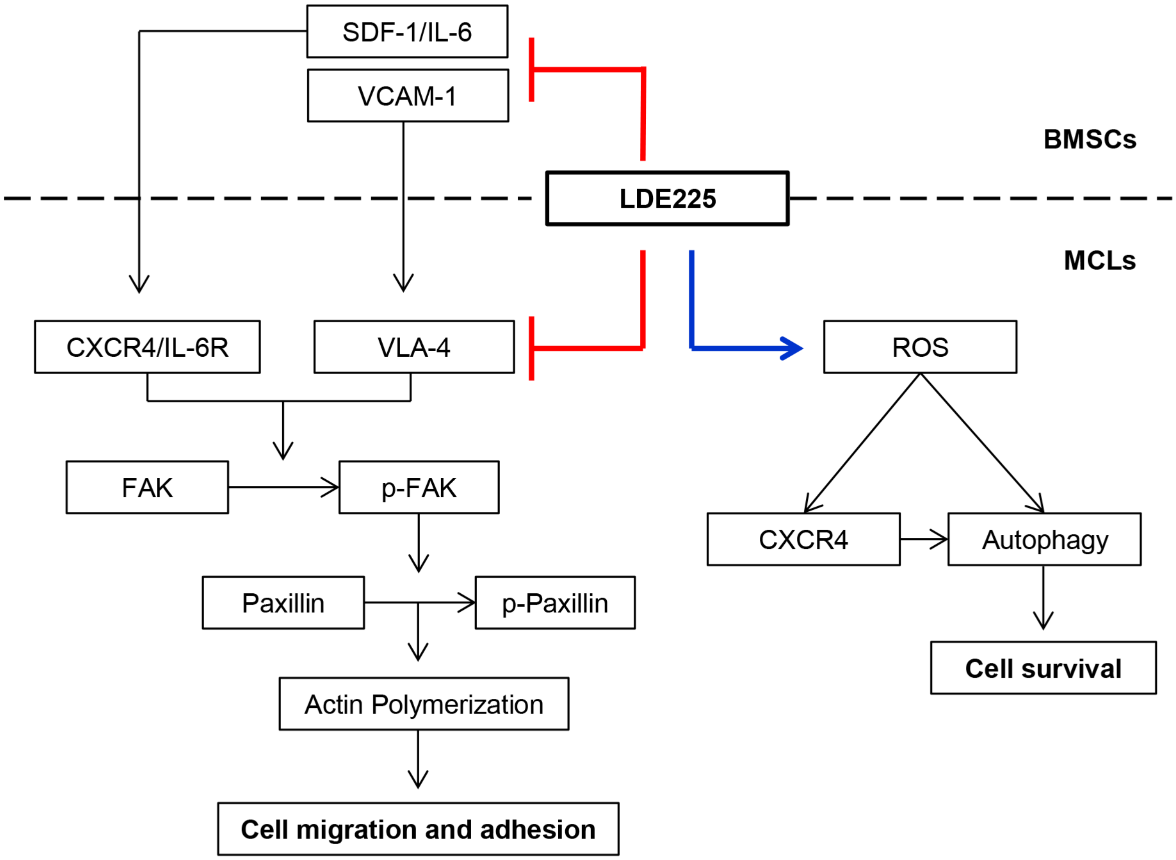

Supplementary Figure S10: Hypothetical model based on the observations made in this study. LDE225 inhibits MCL cell migration and adhesion to stromal cells via VLA-4-mediated inactivation of FAK signaling. LDE225 also disrupt stromal cell production of chemokine SDF-1, cytokine IL-6 and integrin ligand VCAM-1, which in turn further blocks activated signaling from BMSCs. However, ROS produced after LDE225 treatment contributes to CXCR4 upregulation, which in turn stimulates autophagy. Upregulated autophagy benefits MCL cell survival.

Supplementary Table S1: VLA-4 expressions in MCL patients and cell lines with LDE225 treatment

MFI	MCL pt 1	MCL pt 2	SP53	Jeko	REC1
DMSO	43.5	17.9	68.2	44.4	40.9
LDE225 (30 μM)	11.8	4.9	54.8	33.7	30.6
Percentage change	72.9%	72.6%	19.6%	24.1%	25.2%

Supplementary Table S2: CXCR4 expressions in MCL patients and cell lines with LDE225 treatment

MFI	MCL pt 1	MCL pt 2	SP53	Jeko	REC1
DMSO	37.4	67.2	64.0	73.6	34.7
LDE225 (30 μM)	56.7	80.1	76.7	89.1	49.7
Percentage change	51.6%	19.2%	19.8%	21.1%	43.2%

Supplementary Table S3: CXCR4 expressions with pre-treatment of ROS-inhibitor NAC

MFI	MCL pt 1	MCL pt 2	SP53	Jeko	REC1
LDE225 (30 µM)	56.7	80.1	76.7	89.1	49.7
LDE225+NAC	33.0	62.5	61	34.5	33.4
Percentage change	41.8%	22.0%	20.5%	61.3%	32.8%

Supplementary Table S4: CXCR4 expressions with co-treatment of CXCR4-antagonist AMD3100

MFI	MCL pt 1	MCL pt 2	SP53	Jeko	REC1
LDE225 (30 µM)	56.7	80.1	76.7	89.1	49.7
LDE225+AMD3100	3.3	3.2	4.5	7.3	18.2
Percentage change	94.2%	96.0%	94.1%	91.8%	63.4%

Expression characteristics of MCL cells presented as mean fluorescence intensity (MFI) for different molecules.

Supplementary Table S5: Gene-specific primers used for real-time PCR

Gene	Forward primer (5’-3’)	Reverse primer (5’-3’)
Gli1	CCCTTCAAAGCCCAGTACAT	TTTCGAGGCGTGAGTATGAC
Gli2	AAGGAAGATCTGGACAGGGA	TGCTCGTTGTTGATGTGATG
Ptch1	TCATCAGAGTGTCGCACAGA	GCATAGGCGAGCATGAGTAA
FAK	GAAGGCCAATTTGGAGATGT	CACGCTGTCCGAAGTACAGT
Paxillin	TCAGGACAGTGTTGGCTCTC	CGGTCGAGTTCAGAAAGGTT
CXCR4	GGTGGTCTATGTTGGCGTCT	TGGAGTGTGACAGCTTGGAG
VLA-4	TCAAGCATTTATGCGGAAAG	AAGTGGTGGGAATTCCTCTG
SDF-1	CTCAACACTCCAAACTGTGC	CCAGGTACTCCTGAATCCAC
VCAM-1	TTTCTGGAGGATGCAGACAG	TTTAGCTCGGCAAACAAGAA
IL-6	CCAGGAGCCCAGCTATGAAC	CCCAGGGAGAAGGCAACTG
GAPDH	GACAGTCAGCCGCATCTTCT	GCGCCCAATACGACCAAATC
